# Comparative RNA-Seq and Phytochemical Profiling Reveal Year-Dependent Bioactive Variations and Delineate Key Metabolic Pathways in *Polygonatum sibiricum* Red. Rhizome Nodes

**DOI:** 10.3390/ijms27188282

**Published:** 2026-09-17

**Authors:** Chunming Li, Xiaoyu Su, Yaling Yang, Yiwen Cao, Lei Li, Dandan Lu, Yao Sun, Lina Wang, Mengfan Su, Yongliang Yu, Haitao Lu, Zhengwei Tan, Huizhen Liang

**Affiliations:** 1 Institute of Chinese Herbal Medicines, Henan Academy of Agricultural Science, Zhengzhou 450002, Chinahnndlina@163.com (L.W.); sufancy1669@163.com (M.S.); haitaolu26@163.com (H.L.); 2 Provincial Key Laboratory of Conservation and Utilization of Traditional Chinese Medicine Resources, Institute of Chinese Herbal Medicines, Henan Academy of Agricultural Sciences, Zhengzhou 450002, China

**Keywords:** *Polygonatum sibiricum*, rhizome age nodes, transcriptomics, bioactivecomponents, metabolic reprogramming

## Abstract

*Polygonatum sibiricum* Red. is a medicinal and edible homologous plant whose rhizome quality determines its clinical efficacy and commercial value. However, conventional evaluations typically treat the entire rhizome as homogeneous, overlooking the intrinsic heterogeneity along age nodes, and the regulatory mechanisms governing such age-dependent quality variation remain largely unclear. In this study, four-year-old rhizomes were precisely segmented into four age nodes (HJ1–HJ4), and the contents of polysaccharides, flavonoids, and saponins were systematically determined alongside comparative transcriptome sequencing to dissect the age-dependent bioactive variation and underlying molecular mechanisms. The three bioactive components exhibited markedly asynchronous accumulation patterns, with polysaccharides peaking at HJ3, flavonoids at HJ4, and saponins at HJ1. Transcriptomic analysis identified 19,638 differentially expressed genes (DEGs), with KEGG enrichment pointing to starch and sucrose metabolism, flavonoid biosynthesis, and steroid biosynthesis as the pivotal pathways. A coordinated transcriptional reprogramming emerged across development, characterized by early upregulation of backbone-construction genes and progressive activation of modification-and-decoration genes—yet it manifested through pathway-specific regulatory logics, with Mantel tests further confirming that key genes, including *β-amylase3*, *CYP75B11*, and squalene epoxidase (*SQLE*), were significantly correlated with the accumulation of polysaccharides, flavonoids, and saponins, respectively. These findings challenge the conventional assumption that older nodes are universally superior and establish that age-node differentiation reflects fundamental metabolic reprogramming, supporting a node-targeted utilization strategy encompassing HJ3 for polysaccharide-based products, HJ4 for antioxidant applications, HJ1 for saponin extraction, and HJ2 for bulk materials, while providing both mechanistic insights and practical guidance for precision harvesting and quality-oriented management of *P. sibiricum*.

## 1. Introduction

Medicinal plants serve as critical resources for natural bioactive compounds in both traditional and modern medicine [1]. *Polygonatum sibiricum* Red., a perennial medicinal plant belonging to the genus *Polygonatum* within the family Asparagaceae (formerly Liliaceae), is a quintessential representative of medicinal and edible homologous Chinese herbal materials in China [2]. Its dried rhizome is officially documented in the *Pharmacopoeia of the People’s Republic of China* and is recognized for its effects in replenishing qi, nourishing yin, strengthening the spleen, moistening the lungs, and benefiting the kidneys, rendering it widely utilized in traditional Chinese medicine (TCM) clinical formulations and the development of functional health products [3,4]. Modern pharmacological studies have demonstrated that the primary bioactive constituents of *P. sibiricum* include polysaccharides, flavonoids, and steroidal saponins, among other secondary metabolites [5,6,7]. These compounds exhibit stable and significant biological activities, including antioxidant, immunomodulatory, hypoglycemic, cognitive-enhancing, and neuroprotective effects, which directly determine the quality, clinical efficacy, and commercial value of *P. sibiricum* medicinal materials.

The rhizome serves as the primary medicinal organ of *P. sibiricum*. Unlike annual plants, the rhizome of *P. sibiricum* exhibits a distinctive growth pattern, in which one annual node segment is differentiated each year, ultimately forming a chain-like structure composed of segments of varying ages within a single rhizome [8]. In traditional cultivation and clinical practice, only rhizomes of 3–4 years or older are harvested for medicinal use, as it is generally recognized that prolonged growth years are associated with superior medicinal quality, implying a close relationship between secondary metabolite accumulation and the age of individual rhizome nodes [4,9,10]. To date, most studies have focused on comparing the component contents of *P. sibiricum* from different geographical origins or cultivation years and have generally revealed dynamic changes in polysaccharides, saponins, and other indicators with increasing growth years [11,12]. However, the majority of these investigations have treated the entire rhizome as a single unit without precise segmentation according to age nodes, thereby failing to elucidate the patterns of material accumulation at distinct developmental stages within the same rhizome. Although some studies have indicated that rhizome nodes are closely correlated with growth years, and that significant differences in morphology, texture, and chemical composition exist between young and mature nodes, systematic quantitative data regarding the co-accumulation characteristics of polysaccharides, flavonoids, and saponins across different age nodes (1–4 years) remain lacking.

By contrast, transcriptomic studies on *P. sibiricum* have predominantly focused on stress responses, variety comparisons, or tissue-specific differences, whereas systematic investigations addressing the developmental progression of different age nodes and the coordinated regulatory mechanisms of the three major bioactive components remain scarce [13,14,15]. In particular, there is a notable lack of correlative evidence linking the expression of key structural genes with component accumulation, which constrains the molecular-level guidance of precision harvesting and quality-oriented breeding of *P. sibiricum*. In this study, we focused on polysaccharides, flavonoids, and saponins, as these are among the most extensively studied bioactive compound classes consistently associated with the medicinal properties of *P. sibiricum*. To investigate the age-dependent accumulation patterns of these components within the rhizome, four-year-old *P. sibiricum* rhizomes were used as experimental materials, and the age nodes were finely delineated into HJ1–HJ4. The total contents of polysaccharides, flavonoids, and saponins in each age node were systematically determined. Combined with transcriptome sequencing, differential gene expression analysis and functional enrichment were performed, with a particular focus on elucidating the temporal expression patterns of three major pathways, namely starch and sucrose metabolism, flavonoid biosynthesis, and steroidal saponin biosynthesis. Furthermore, correlation analyses were conducted to reveal the associations between gene expression and secondary metabolite accumulation, thereby clarifying the dynamic accumulation patterns and molecular regulatory mechanisms of the principal bioactive components across different age nodes. These findings are expected to provide both theoretical foundations and technical support for superior-quality germplasm screening, node-based grade classification, and comprehensive development and utilization, as well as improved cultivation, harvesting, and breeding practices of *P. sibiricum*.

## 2. Results

### 2.1. Differences in Bioactive Content in Different Age-Node Rhizomes of P. sibiricum

**Figure 1:**
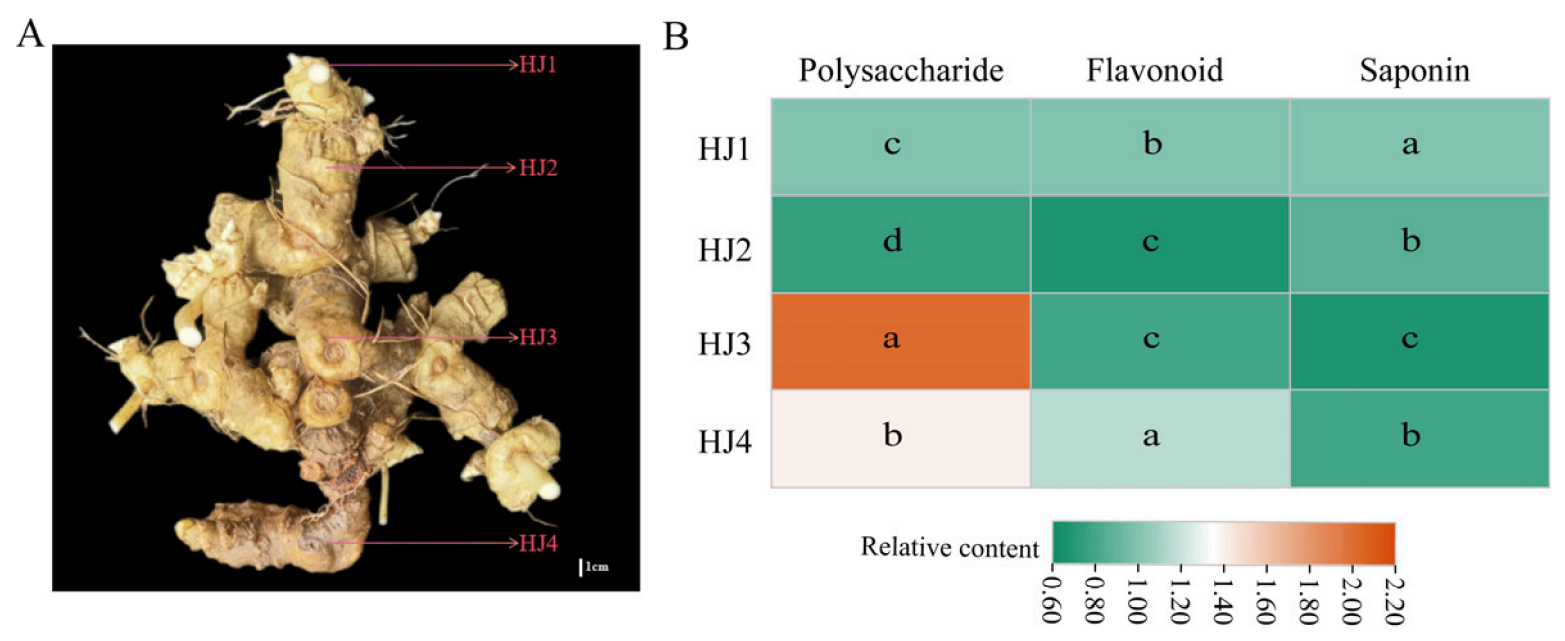
Phenotypic characteristics and major active ingredient contents in different age-node rhizomes of *P. sibiricum*. (**A**) Schematic representation of the different age-node rhizome positions of *P. sibiricum* (HJ1, HJ2, HJ3, and HJ4 represent 1-, 2-, 3-, and 4-year-old rhizomes, respectively; scale bar = 1 cm); (**B**) heatmap showing the relative contents of polysaccharides, flavonoids, and saponins (mean ± SD, *n* = 3). Different lowercase letters above the bars indicate significant differences based on one-way ANOVA (*p* value < 0.05).

As shown in Figure 1A, the rhizome morphology of *P. sibiricum* differed markedly across age nodes (HJ1–HJ4), with color progressively deepening with age. The contents of polysaccharides, flavonoids, and saponins also varied significantly among the four age nodes (Figure 1B, Table S1). The 3-year-old rhizomes (HJ3) had the highest polysaccharide content (237.9 mg/g DW), more than double that of HJ1. Flavonoids were most abundant in HJ4 (0.24 mg/g DW), with no significant difference between HJ2 and HJ3. Saponin content was highest in HJ1 (9.98 mg/g DW), decreased over the first three years, and then increased substantially in HJ4.

### 2.2. Transcriptomic Analysis of HJ Across Different Plant Age Sections

**Figure 2:**
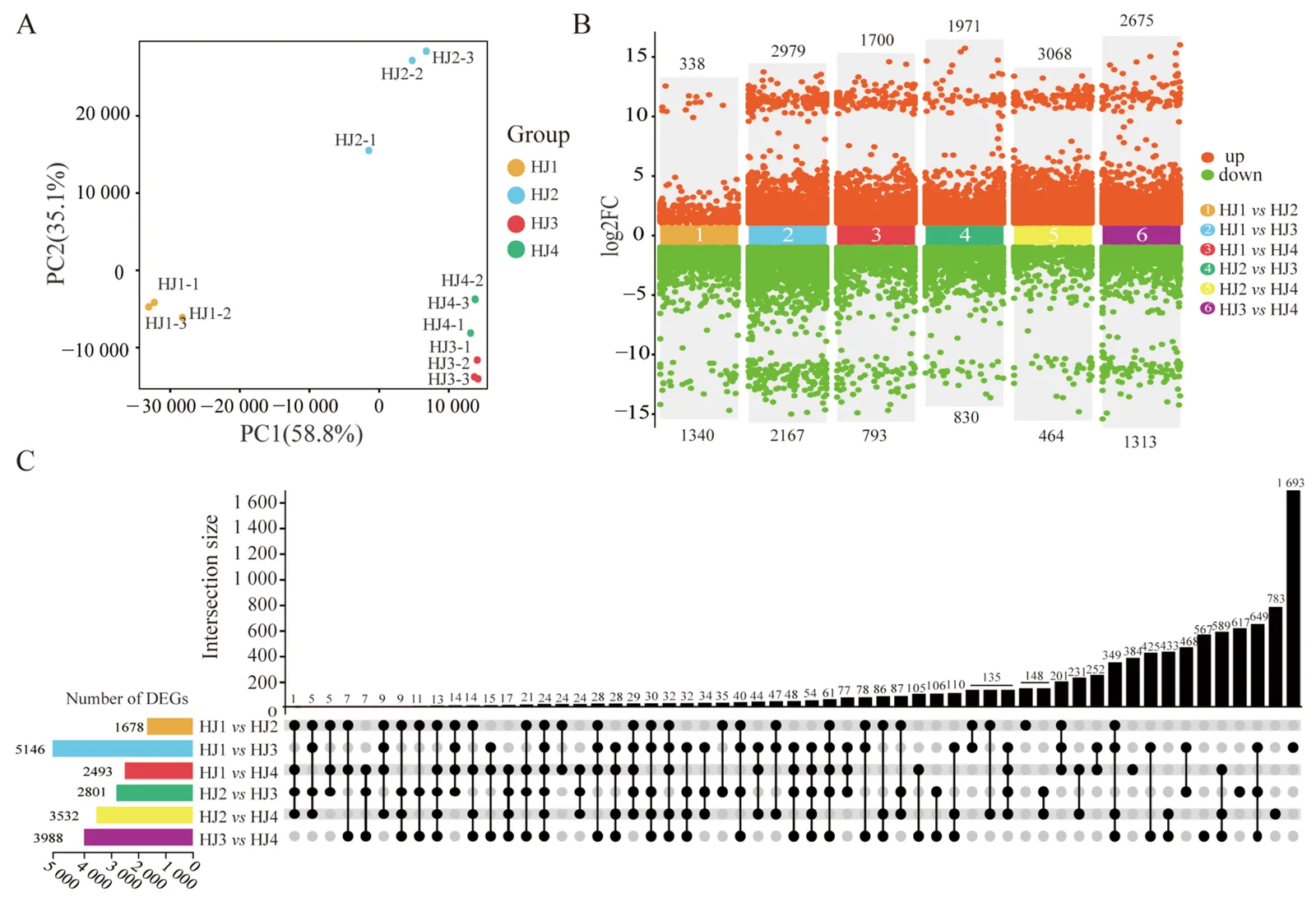
Transcriptomic profiling of different age-node rhizomes of *P. sibiricum*. (**A**) Principal component analysis (PCA) score plot of 12 independent samples; (**B**) statistics on the number of up- and down-regulated DEGs in different comparison groups; (**C**) UpSet diagram of DEGs in different comparison groups.

To elucidate the molecular regulatory mechanisms underlying the differential accumulation of bioactive compounds in different age-node rhizomes of *P. sibiricum*, a de novo transcriptome sequencing analysis was performed in this study. Raw sequencing data were obtained for 12 samples, and after quality filtering, each sample yielded over 5.183 GB of high-quality clean reads (Table S2). The sequencing data quality was high, with Q30 base percentages across all samples ranging from 93.66% to 94.34% and averaging 94.05%, and GC content averaging 46.45%, indicating reliable data for subsequent analyses. Subsequently, to evaluate the overall transcriptional variation among different age-node rhizome samples, principal component analysis (PCA) was conducted. As shown in Figure 2A, the first principal component (PC1) and the second principal component (PC2) explained 58.8% and 35.1% of the total variance, respectively, with a cumulative contribution rate of 93.9%. In terms of sample distribution, clear inter-group differences were observed, along with good intragroup reproducibility, confirming the reliability of the transcriptome data.

Differential expression analysis was subsequently performed, and pairwise comparisons of the transcriptomic profiles among different age-node rhizomes identified a total of 19,638 differentially expressed genes (DEGs) (Figure 2B). Among all comparison groups, the HJ2 vs. HJ3 comparison exhibited the largest number of DEGs, with 2979 upregulated and 2167 genes. To visually depict the intersection relationships of DEGs across multiple comparison groups, an UpSet plot was generated (Figure 2C). Notably, the HJ1 vs. HJ3 comparison group contained the highest number of unique DEGs (1693 genes), suggesting that substantial transcriptional regulatory changes may occur between the 1-year-old and 3-year-old nodes as the rhizome ages. In contrast, only 24 DEGs were commonly identified across all six comparison groups. These shared genes likely represent a core set of genes closely associated with age-related changes during rhizome development in *P. sibiricum*, exhibiting consistent expression differences across different age nodes.

**Figure 3:**
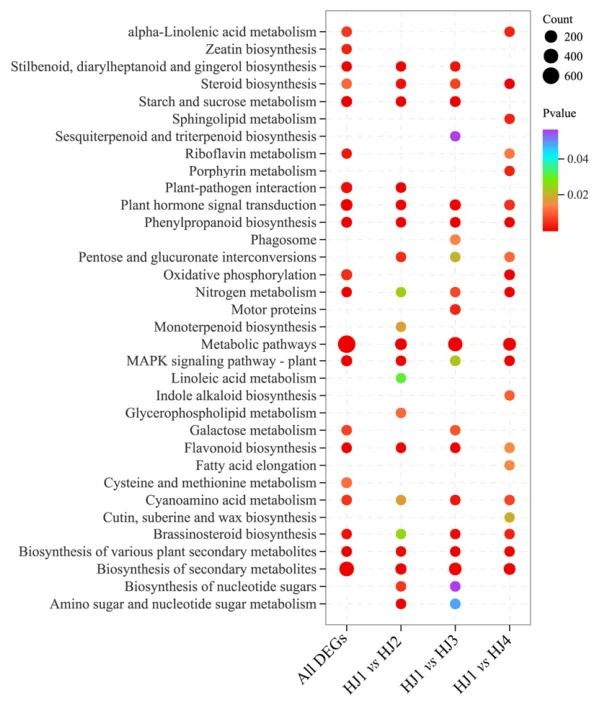
KEGG pathway enrichment analysis of DEGs in different age-node rhizomes of *P. sibiricum*.

Furthermore, three differential gene sets obtained from pairwise comparisons against the 1-year-old node (HJ1) as the reference, namely HJ1 vs. HJ2, HJ1 vs. HJ3, and HJ1 vs. HJ4, were selected for enrichment analysis. Kyoto Encyclopedia of Genes and Genomes (KEGG) enrichment analysis indicated that the DEGs were significantly enriched in multiple metabolic pathways, including starch and sucrose metabolism, steroid biosynthesis, and flavonoid biosynthesis. These biosynthetic processes are closely associated with the formation of bioactive compounds in *P. sibiricum* (Figure 3).

### 2.3. Expression Dynamics of Starch and Sucrose Metabolism Pathway Genes Across Different Age Nodes of P. sibiricum

**Figure 4:**
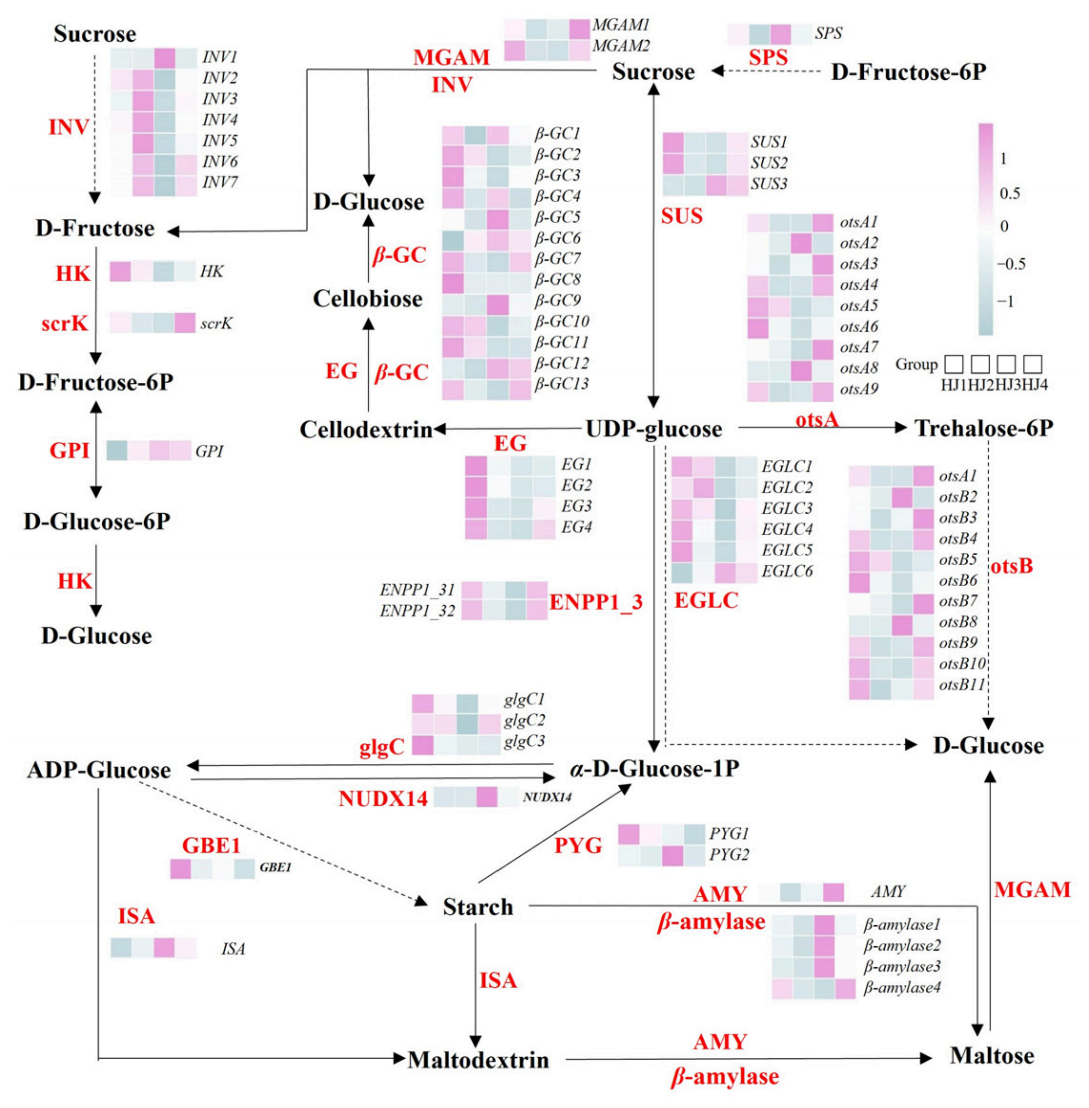
Starch and sucrose metabolism pathway and expression profiles of related enzyme-encoding genes in different age-node rhizomes of *P. sibiricum*. Schematic representation of the starch and sucrose metabolism pathway, with red letters indicating the DEGs at different developmental stages. Heatmap showing the expression patterns of these enzyme-encoding genes across the four age-node rhizome sections. The black solid line arrows represent direct effects, and the dashed line arrows represent indirect effects.

Sucrose serves as the primary form of carbon transport, and its degradation and conversion generate a series of activated glycosyl donors, providing a critical substrate foundation for downstream polysaccharide biosynthesis [16]. During the continuous developmental progression from the 1-year-old to the 4-year-old nodes of *P. sibiricum* rhizomes, a total of 74 DEGs were identified within the starch and sucrose metabolism pathway (map00500) (Table S3). Heatmap analysis of the expression profiles (Figure 4) revealed that the majority of DEGs exhibited a downregulated then upregulated expression pattern, involving genes associated with sucrose hydrolysis (*hexokinase*, *scrK*, and *SUS*), the rate-limiting step of starch biosynthesis (*glgC*), starch degradation (*AMY*), and sucrose synthesis (*SPS*). A subset of genes displayed a consistently downregulated trend, including those involved in polysaccharide depolymerization (*β-GC*) and a key enzyme for amylopectin branch formation (*GBE1*), whereas other genes, such as β-amylase, exhibited an initially upregulated followed by expression pattern. These expression dynamics reflect the temporal regulation of carbon partitioning and polysaccharide metabolism in *P. sibiricum* rhizomes across increasing age nodes. At the 1-year-old node, which represents the early stage of rhizome formation, the relatively low expression of sucrose hydrolysis- and starch degradation-related genes may facilitate the initial accumulation of carbon sources. During the 2- to 3-year-old nodes, corresponding to the critical period of rapid rhizome growth and material accumulation, the expression of genes involved in starch biosynthesis and sucrose conversion progressively increased, accompanied by a marked enhancement of polysaccharide anabolic metabolism. At the 4-year-old node, the rhizome approached maturity, as reflected by the declining expression of certain degradation-related genes, stabilization of polysaccharide accumulation, and reduced metabolic activity. Collectively, the temporal expression patterns of starch and sucrose metabolism-related genes across different age nodes of *P. sibiricum* rhizomes coordinately regulate the generation and conversion of glycosyl donors, thereby providing a molecular basis for the dynamic accumulation of polysaccharides during rhizome development.

### 2.4. Expression Dynamics of Flavonoid Biosynthesis Pathway Genes Across Different Age Nodes of P. sibiricum

**Figure 5:**
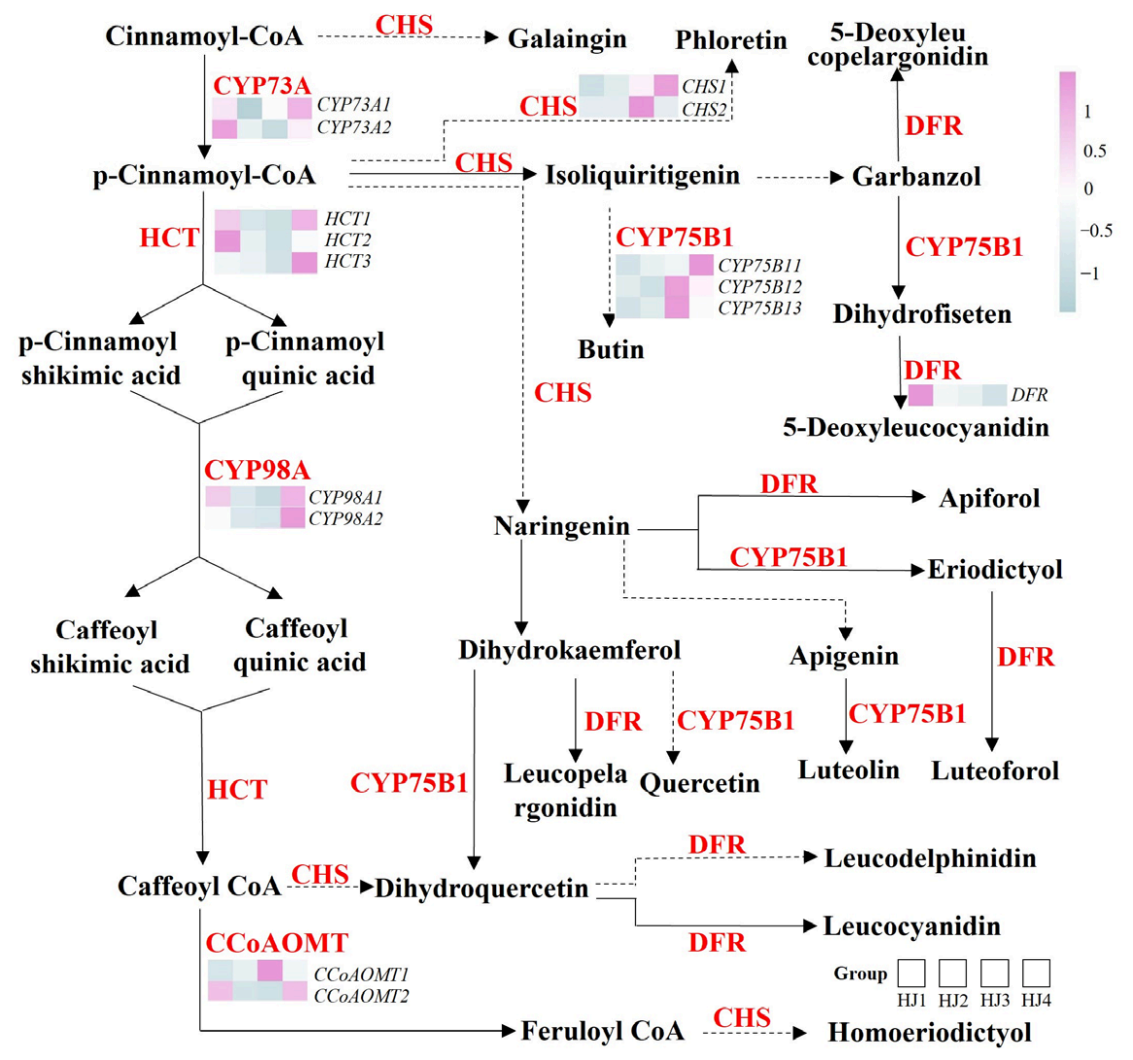
Flavonoid biosynthesis pathway and expression profiles of related enzyme-encoding genes in different age-node rhizomes of *P. sibiricum*. Schematic representation of the starch and sucrose metabolism pathway, with red letters indicating the DEGs at different developmental stages. Heatmap showing the expression patterns of these enzyme-encoding genes across the four age-node rhizome sections. The black solid line arrows represent direct effects, and the dashed line arrows represent indirect effects.

As shown in Figure 5 and Table S4, a total of 14 DEGs were identified within the flavonoid biosynthesis pathway (map00941) across different age-node samples of *P. sibiricum*. The heatmap revealed that genes involved in upstream skeleton biosynthesis, including members of the CHS family (*CHS1* and *CHS2*) and the CYP73A family (*CYP73A1* and *CYP73A2*), were highly expressed at the HJ1 node and exhibited a generally downregulated trend with increasing age nodes. In contrast, downstream modification and reduction genes, including the HCT family (*HCT1*, *HCT2*, and *HCT3*), the CYP75B1 family (*CYP75B11*, *CYP75B12*, and *CYP75B13*), bifunctional dihydroflavonol 4-reductase/flavanone 4-reductase (*DFR*), and the CCoAOMT family (*CCoAOMT1* and *CCoAOMT2*), displayed relatively elevated expression levels from HJ2 to HJ4. These expression patterns suggest that the HJ1 node primarily activates upstream phenylpropanoid pathway and skeleton biosynthesis genes to accumulate precursors for flavonoid synthesis. As development progresses into the HJ2 to HJ4 nodes, the upregulation of genes involved in hydroxylation, reduction, and methylation modifications drives the conversion of intermediates toward final products, including flavones, flavonols, and anthocyanins.

### 2.5. Expression Dynamics of Steroid Biosynthesis Pathway Genes Across Different Age Nodes of P. sibiricum

As important secondary metabolites in *P. sibiricum*, saponins are biosynthesized starting from farnesyl pyrophosphate (Farnesyl-PP) through the squalene synthesis pathway to generate squalene, which is subsequently catalyzed by squalene epoxidase SQLE to form (S)-2,3-oxidosqualene, thereby establishing the precursor foundation for saponin backbone construction [17,18]. Through intermediates such as cycloartenol and lanosterol, a series of 1,4-demethylation, double-bond isomerization, and backbone modifications are catalyzed by key enzymes including CYP51, ERG27, EBP, and MCM [19]. Subsequently, methyltransferases (SMT1) and oxidases (SMO1, SMO2) mediate carbon-chain alkylation and hydroxylation reactions to progressively generate phytosterols and saponin precursors, including stigmasterol, campesterol, and sitosterol [20]. These precursors ultimately undergo acylation and esterification modifications, catalyzed by lipases of the LIPA family, to form cholesterol esters and other terminal products, constituting the complete biosynthetic pathway of saponins in *P. sibiricum*.

**Figure 6:**
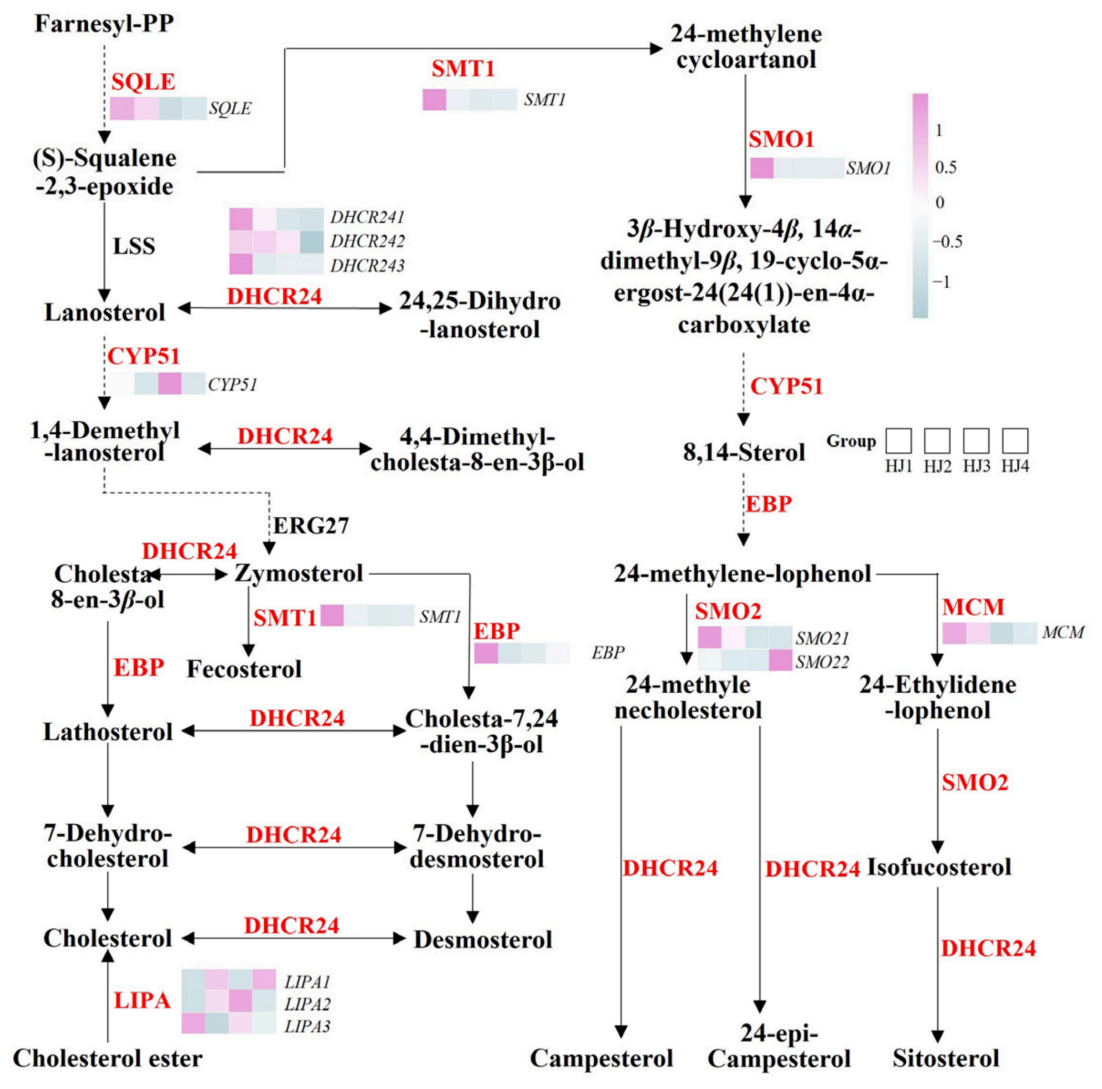
Steroid biosynthesis pathway and expression profiles of related enzyme-encoding genes in different age-node rhizomes of *P. sibiricum*. Schematic representation of the starch and sucrose metabolism pathway, with red letters indicating the DEGs at different developmental stages. Heatmap showing the expression patterns of these enzyme-encoding genes across the four age-node rhizome sections. The black solid line arrows represent direct effects, and the dashed line arrows represent indirect effects.

Across different age-node samples of *P. sibiricum*, a total of 14 DEGs were identified within the steroid pathway (map00100) (Figure 6 and Table S5). Core functional genes, including *SQLE*, *SMT1*, *SMO1*, *EBP*, and members of the *DHCR24* family, exhibited distinct expression divergence across different age nodes. The heatmap revealed that certain genes were significantly upregulated at the HJ1, with expression progressively declining from HJ2 to HJ4. In contrast, downstream modification genes, such as *SMO2* and members of the *LIPA* family, displayed relatively elevated expression levels during the HJ2–HJ4. These expression patterns suggest that the HJ1 is predominantly committed to initiating upstream precursor synthesis and backbone construction, with high expression of relevant biosynthetic genes facilitating the accumulation of saponin precursors. From HJ2 to HJ4, the downregulation of upstream biosynthesis genes, coupled with the upregulation of downstream modification and conversion genes, drives the transformation of sterol intermediates toward terminal saponin products. Collectively, these findings indicate that saponin biosynthesis in *P. sibiricum* is coordinately regulated by multiple genes, and the temporal up- and down-regulation patterns of key pathway genes are closely associated with the accumulation of secondary metabolites and physiological developmental progression across different age nodes.

### 2.6. Correlation Analysis Between DEGs and Polysaccharides, Flavonoids, and Saponins

**Figure 7:**
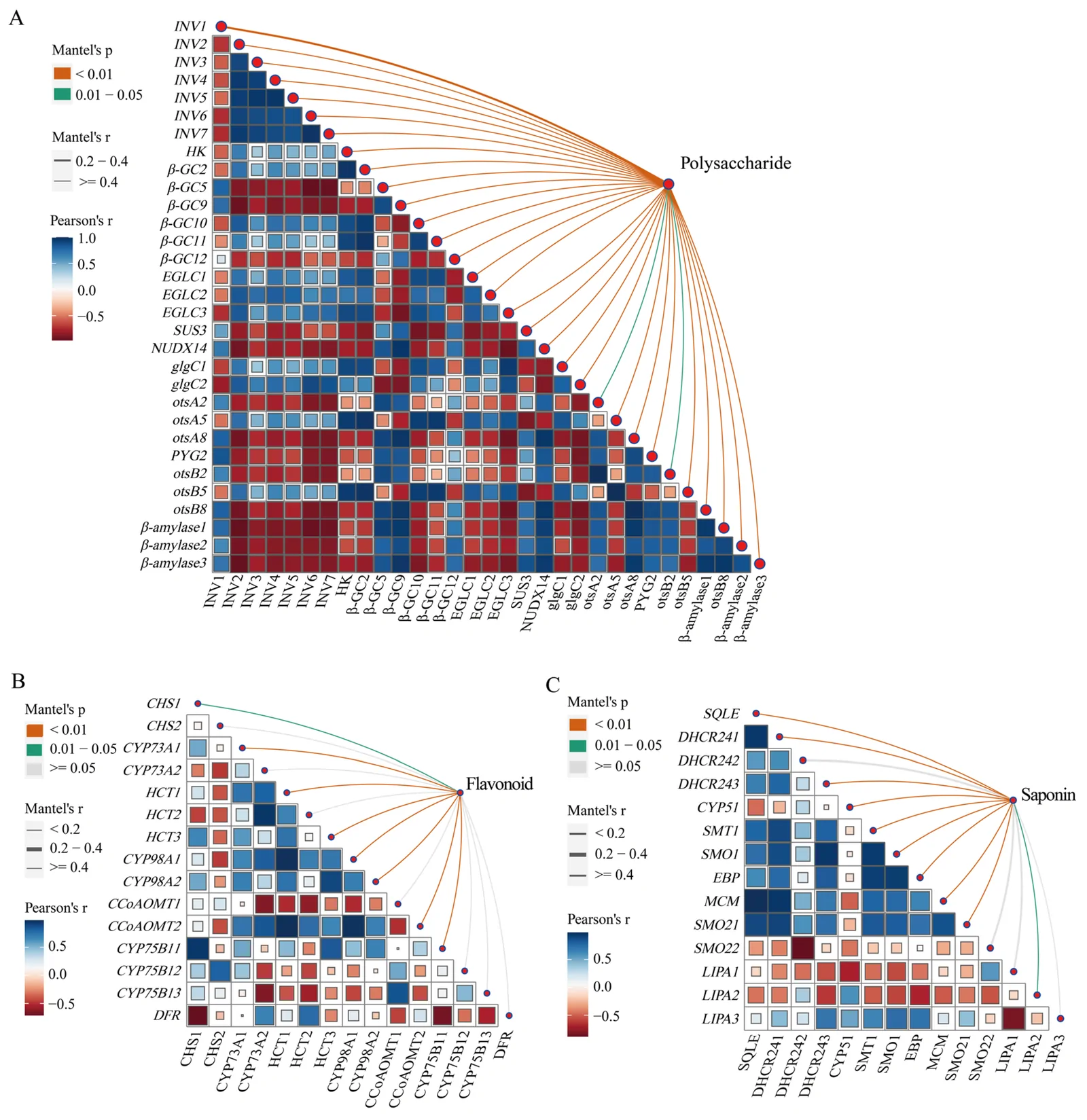
Correlations between DEGs and major active ingredient contents in different age-node rhizomes of *P. sibiricum* based on Mantel tests. (**A**–**C**) Mantel test correlations between the expression profiles of DEGs and polysaccharide contents, flavonoid contents, and steroidal saponin contents, respectively.

Mantel tests were separately performed between the content matrices of polysaccharides, flavonoids, and saponins across different age nodes and those of DEGs involved in their respective biosynthetic pathways (Tables S6–S8). As shown in Figure 7, all DEGs within the starch and sucrose metabolism pathway exerted a significant influence on polysaccharide accumulation in *P. sibiricum* (Mantel’s r ≥ 0.4, Mantel’s *p* value < 0.05), among which *β-amylase3*, *INV2*, and *β-GC9* ranked the top three with the most significant positive correlations (Mantel’s r ≥ 0.8, Mantel’s *p* value < 0.05). Regarding flavonoid accumulation across different age nodes, a subset of DEGs, including *CYP73A1*, *HCT1*, *HCT3*, *CYP98A1*, *CYP98A2*, *CCoAOMT2*, and *CYP75B11*, exhibited significant positive correlations with flavonoid biosynthesis (Mantel’s r > 0.5, Mantel’s *p* value < 0.05). For saponin accumulation, genes including *SQLE*, *DHCR241*, *CYP51*, *SMT1*, *SMO1*, *MCM*, and *SMO21* displayed significant positive correlations with steroidal saponin accumulation (Mantel’s r > 0.5, Mantel’s *p* value < 0.05). Pearson correlation analysis among genes revealed the presence of positive, negative, or weak synergistic expression relationships within each of the three pathways, with the highest degree of synergy observed among genes in the polysaccharide pathway.

### 2.7. Quantitative Real-Time PCR (RT-qPCR) Validation of RNA-Seq Data

**Figure 8:**
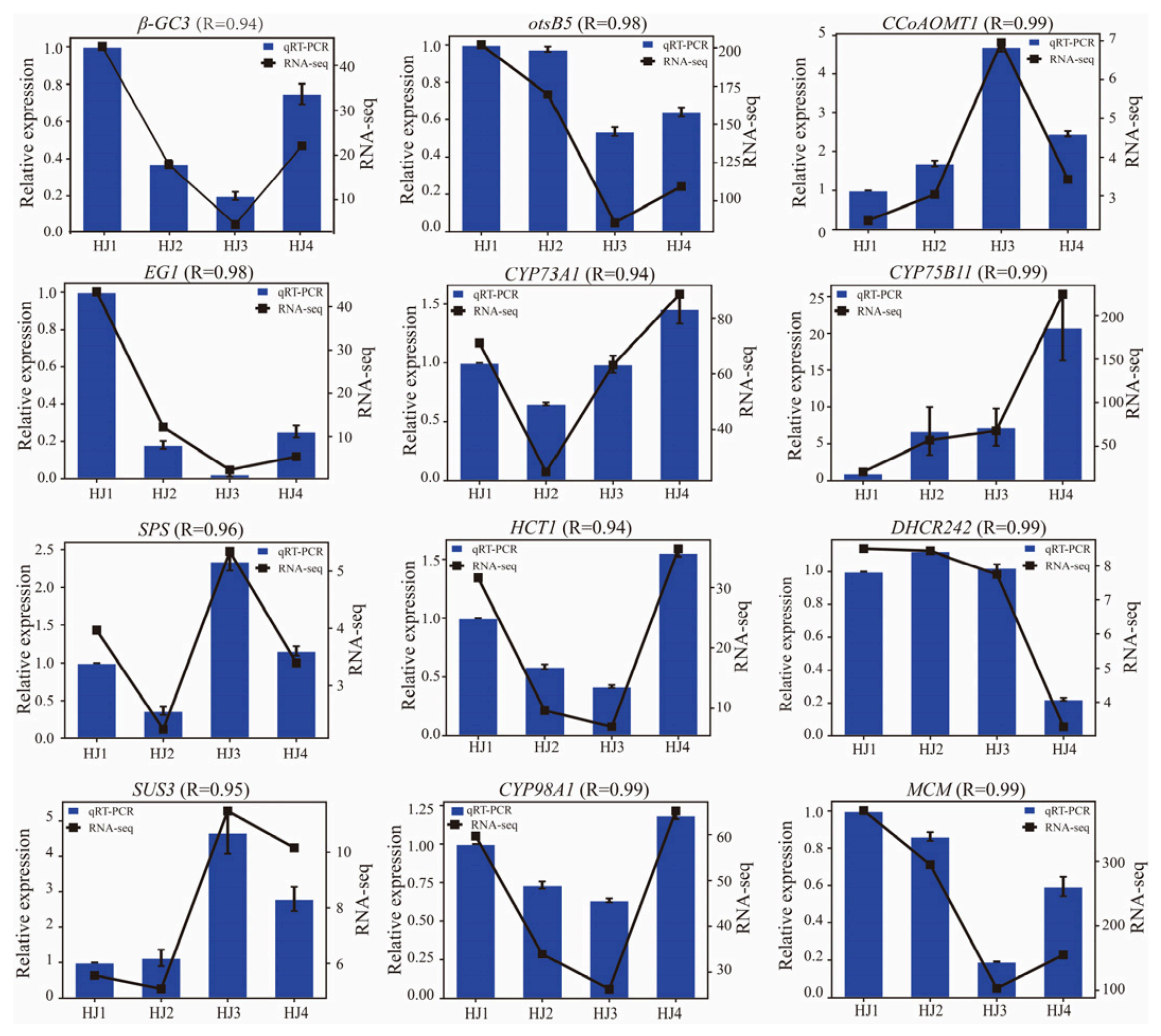
RT-qPCR validation of 12 selected genes from starch and sucrose metabolism, flavonoid biosynthesis, and steroidal saponin biosynthesis pathways. Pearson correlation analysis between the two datasets yielded correlation coefficients ranging from 0.94 to 0.99 across the 12 validated genes.

To validate the expression patterns of the DEGs that had been screened from the starch and sucrose metabolism, flavonoid biosynthesis, and steroidal saponin biosynthesis pathways, we performed RT-qPCR analysis on 12 selected genes. The expression trends were found to be broadly comparable to the FPKM values derived from RNA-seq (Figure 8). To further quantitatively assess the reliability of the RNA-seq data, we performed Pearson correlation analysis between the RNA-seq (FPKM) and RT-qPCR (relative expression) data across the 12 validated genes. The correlation coefficients ranged from 0.94 to 0.99, indicating a strong and significant positive correlation between the two platforms (Figure 8). These results collectively confirm the robustness of our transcriptomic profiling and support the involvement of these DEGs in the regulation of carbohydrate metabolism, flavonoid biosynthesis, and saponin accumulation across different age-node rhizomes of *P. sibiricum*.

## 3. Discussion

### 3.1. Developmental Stage-Specific Metabolic Trade-Offs Shape Bioactive Profiles in P. sibiricum Rhizome Nodes

Most previous evaluations of *P. sibiricum* quality have treated the entire rhizome as a homogeneous tissue, typically comparing samples from different geographical origins or overall cultivation years. While such approaches have established a general trend of increasing bioactive content with plant age, they inevitably obscure the intrinsic heterogeneity along the rhizome axis. In this study, by precisely segmenting 4-year-old rhizomes into four individual age nodes (HJ1–HJ4), we demonstrate that the accumulation patterns of the three major bioactive components, including polysaccharides, flavonoids, and saponins, are markedly asynchronous. Polysaccharides peaked at HJ3, flavonoids reached their maximum at HJ4, and saponins were highest in the youngest node (HJ1). These results challenge the conventional assumption that “older is always better” and underscore the necessity of node-resolved evaluation.

This asynchrony likely reflects a developmental shift in metabolic prioritization. Young nodes (HJ1) may invest more in saponin-based chemical defense to protect actively dividing tissues, while mature nodes (HJ3–HJ4) gradually switch to storage-type polysaccharides and senescence-associated antioxidant flavonoids—a strategy analogous to the “growth-defense trade-off” observed in other perennial medicinal plants [21]. This developmental shift in metabolic prioritization—from early investment in defensive specialized metabolites to later accumulation of storage compounds and antioxidants—has been documented in diverse perennial species, including the perennial herb *Aristolochia contorta*, which transitions from aristolochic acid accumulation in young plants to alternative alkaloids in maturity [22]. In medicinal plants such as *Panax ginseng*, jasmonate-mediated signaling hyperactivation diverts carbon flux from growth toward defensive ginsenoside biosynthesis at the 2,3-oxidosqualene branch point, exemplifying the growth-defense antagonism [23]. Notably, recent multi-omics analyses in *Polygonatum* ecotypes have directly revealed contrasting carbon allocation strategies: a “growth-type” pattern favoring biomass and polysaccharide accumulation versus a “defense-type” pattern channeling carbon toward shikimic acid-derived secondary metabolites at the expense of growth [24]. The progressive darkening of rhizome color from HJ1 to HJ4 further supports this interpretation, as pigmentation changes often accompany tissue maturation and secondary metabolite redistribution. Similar patterns have been observed in other rhizomatous species; for instance, the rhizome of lotus (*Nelumbo nucifera* Gaertn.) shifts from pale white to light yellow with increasing browning degree during growth, accompanied by significant accumulation of total phenolics, flavonoids, and enhanced antioxidant capacity [25]. Likewise, in *Astragalus membranaceus* var. *mongholicus* (Bunge) P. K. Hsiao, red-stemmed plants consistently accumulate higher levels of isoflavones than green-stemmed plants across all ages (1–5 years), with contents in both types increasing progressively with age and peaking in the fourth year [26]. These observations suggest that the color darkening from young to mature nodes may reflect underlying shifts in secondary metabolite profiles during rhizome development.

### 3.2. Coordinated Transcriptional Reprogramming Underlies the Differential Accumulation of Bioactive Compounds Across Age Nodes in P. sibricum Rhizomes

Polysaccharides are the hallmark bioactive constituents of *P. sibiricum* [27], and their accumulation is intimately linked to carbon partitioning via the starch and sucrose metabolism pathway [28,29]. Our transcriptomic data identified 74 DEGs within this pathway, with expression patterns that dynamically shifted across HJ1–HJ4. At HJ2–HJ3, the critical phase of rapid rhizome expansion, we observed significant upregulation of genes encoding *SUS*, the rate-limiting enzyme of *glgC*, and starch branching enzymes (*GBE1*), coinciding with the peak polysaccharide content at HJ3. This temporal pattern is consistent with the well-established role of SUS and glgC as key determinants of carbon flux toward storage polysaccharides in other rhizomatous plants [30,31] and aligns with previous findings in *P. sibiricum*, where polysaccharide accumulation was most active during the rapid biomass increase phase [32]. Conversely, at HJ1, the relatively low expression of sucrose hydrolysis- and starch degradation-related genes, combined with elevated expression of certain *INV2* and *β-amylase3*, suggests a distinct metabolic state favoring soluble sugar mobilization rather than polymer storage. This inverse relationship between invertase/β-amylase expression and polysaccharide accumulation mirrors the “growth-storage” metabolic switch documented in *Polygonatum odoratum* (Mill.) Druce and other perennial medicinal plants, where young tissues prioritize soluble sugars for immediate metabolic demands, while mature tissues shift toward storage-type polysaccharides [33]. Mantel test correlations further confirmed that *β-amylase3*, *INV2*, and *β-GC9* exhibited the strongest positive associations with polysaccharide content (Mantel’s r ≥ 0.8, *p* value < 0.05), indicating that these genes may serve as molecular markers for polysaccharide accumulation. The progressive downregulation of degradation-related genes at HJ4, coupled with stabilized anabolic gene expression, likely reflects the transition of the rhizome into a relatively quiescent storage state. This pattern parallels the “source-sink transition” described in *Polygonatum cyrtonema* hua, where older rhizome nodes gradually shift from active metabolism to a storage-dominated state as they approach maturity [34]. Collectively, these results provide a mechanistic framework connecting carbon metabolic gene expression to the temporal dynamics of polysaccharide storage in *P. sibiricum* rhizomes.

Flavonoid and saponin biosynthesis represent two specialized metabolic routes that, despite their distinct chemical backbones, share a striking “early skeleton–late decoration” regulatory logic. For flavonoids, upstream core enzymes of the phenylpropanoid pathway, including chalcone synthase (CHS1, CHS2) and cinnamate 4-hydroxylase (CYP73A1, CYP73A2), were highly expressed at HJ1 but progressively declined in older nodes. By contrast, downstream modification genes, such as HCT family members, CYP75B1 (CYP75B11–B13), DFR, and CCoAOMT family members, exhibited elevated expression from HJ2 to HJ4. This expression pattern aligns with the well-established regulatory logic of the flavonoid pathway in model plants, where different R2R3-MYB transcription factors activate early and late biosynthetic steps, respectively [35], and mirrors the developmental expression dynamics observed in other species, such as the early upregulation of *CHS* and *CHI* during the vigorous growth stage of tobacco [36] and the peak of *CHS* expression at the early budding stage of lotus [37], indicating that HJ1 primarily generates the flavonoid skeleton precursors, whereas HJ2–HJ4 drive the conversion of these intermediates into hydroxylated, methylated, and reduced end products.

A similar but distinct pattern was observed for saponin biosynthesis. Genes responsible for upstream squalene synthesis and cyclization—including *SQLE*, *SMT1*, *SMO1*, *EBP*, and *CYP51*—were significantly upregulated at HJ1, which aligns with the highest saponin content measured at this node. As the rhizome aged into HJ2–HJ4, the expression of these upstream biosynthetic genes declined, whereas downstream modification genes such as *SMO2* and *LIPA* family members were relatively elevated. The concept of an “upstream skeleton formation and downstream modification” framework is a recognized principle in the biosynthesis of triterpenoid saponins, and our findings provide temporal gene expression evidence supporting this model in *P. sibiricum*. Interestingly, studies in other medicinal plants have revealed diverse regulatory strategies: in *Ophiopogon japonicus*, for example, the content of saponins increased from the first to the third year, while the expression of related genes paradoxically decreased [38], suggesting that post-transcriptional or translational regulation may play a dominant role in certain contexts—a scenario that contrasts with the transcriptional coordination observed in our study. This coordinated shift suggests that saponin precursor backbones are predominantly laid down at the early developmental stage, while later nodes undergo structural modifications that may generate more diverse or stable saponin variants. Mantel tests further validated that *SQLE*, *DHCR241*, *CYP51*, and *SMT1* were among the genes most positively correlated with saponin accumulation. The strong correlation of these specific upstream genes with saponin content is consistent with the recognized role of *SQLE* as a rate-limiting enzyme and *SMT1* as a key determinant of sterol side-chain diversity, further underscoring their importance as potential targets for metabolic engineering or molecular breeding in *P. sibiricum*.

### 3.3. Node-Based Classification Enables Precision Utilization of P. sibiricum Rhizomes

Our multi-component, transcriptome-integrated assessment reveals that no single node excels across all bioactive categories; instead, each age node exhibits a distinct chemical profile that confers unique strengths for specific applications. This refined view is partially consistent with earlier reports on *P. sibiricum* and related *Polygonatum* species. For instance, Zhang et al. reported that polysaccharides in 1- to 3-year-old nodes remained relatively stable before declining at 6 years, a trend that aligns with our observation of peak polysaccharide accumulation at HJ3 [32]. However, our findings present a more nuanced pattern than the ranking proposed by Chen et al. for *P. cyrtonema* (2-year > 1-year > 3-year > 4-year), which was based on a limited set of physicochemical indices [34]. This discrepancy likely arises from species-specific differences (*P. sibiricum* vs. *P. cyrtonema*), as well as the broader range of quality attributes captured by our expanded analytical framework.

Such age-dependent asynchronous accumulation of bioactive components is not unique to *Polygonatum*; similar phenomena have been observed in other medicinal perennials. In *Panax sokopayensis*, different types of ginsenosides exhibited distinct accumulation peaks across 5- to 20-year-old rhizomes, with protopanaxadiol-type saponins peaking in 10-year-old samples, while protopanaxatriol-type saponins showed different temporal patterns [39]. These parallels reinforce that the asynchronous patterns observed in *P. sibiricum* reflect a common phenomenon, supporting the general applicability of node-based classification.

This refined understanding carries direct implications for harvesting and processing practices. The traditional “bulk harvest” approach, which processes entire rhizomes indiscriminately, inevitably mixes nodes with divergent chemical profiles, leading to batch-to-batch variability and suboptimal utilization of each node’s unique strengths. The concept of node- or age-based differential utilization is not without precedent in medicinal plant research. For instance, in *Astragalus membranaceus* var. *mongholicus*, stem color has been proposed as a visible phenotypic reference for early-stage germplasm selection, as red-stemmed plants consistently accumulate higher levels of bioactive isoflavones than their green-stemmed counterparts across all ages [26]. Similarly, in *Panax ginseng*, ginsenoside profiles differ markedly between different parts of the same root (main root, lateral root, and rhizome), leading to differential utilization—for example, the rhizome (root head) is often specifically processed for its higher ginsenoside Rg1 and Rb1 content [40]. However, these approaches typically rely on single traits or limited markers, whereas our integrated chemical–transcriptional framework simultaneously considers growth, nutritional, and medicinal profiles, offering a more comprehensive basis for node-targeted utilization.

Based on this framework, we propose a rational utilization strategy by matching HJ3 (peak polysaccharides) to polysaccharide-based pharmaceuticals, HJ4 (rich in flavonoids and phenolics) to antioxidant-enriched functional foods or cosmetics, HJ1 (highest saponins) to saponin extraction, and HJ2 (maximal biomass) to bulk materials or feed additives where specific phytochemical profiles are less critical. This age-node-specific differentiation provides a scientific basis for shifting from “mixed harvesting and mixed use” to a refined, value-added system that maximizes both the therapeutic and economic potential of *P. sibiricum* cultivation.

### 3.4. Limitations and Future Perspectives

Several caveats should be acknowledged when interpreting our results. First, this study was conducted with a single genotype of *P. sibiricum* cultivated at one location (Henan, China) and harvested at a single time point (4 years). The extent to which the observed age-node expression patterns and accumulation trends are conserved across different cultivars, edaphic and climatic conditions, and harvest years remains to be investigated, and the generalizability of our findings across these variables has not yet been established. Future studies incorporating multiple varieties, diverse production regions, and varied cultivation durations will be essential to determine whether the asynchronous accumulation patterns observed here represent a conserved developmental feature of *Polygonatum* rhizomes. Second, although our transcriptomic correlations strongly implicate specific genes in the regulation of bioactive compound accumulation, these associations are correlative rather than causal. Further functional validation, such as gene overexpression, VIGS (virus-induced gene silencing), or CRISPR-Cas9-based mutagenesis, is required to establish the regulatory roles of these candidate genes. Third, the phytochemical analysis in this study relied on global colorimetric and spectrophotometric assays for the quantification of total polysaccharides, flavonoids, and saponins. While these methods are widely employed for routine quality evaluation and provide valuable comparative data across samples, they lack the specificity to resolve individual compound profiles and are susceptible to potential matrix interference from crude rhizome extracts. Therefore, our conclusions regarding chemical differences among age nodes are drawn at the class level rather than at the level of specific marker compounds. Future studies employing advanced chromatographic techniques, such as HPLC or UPLC-MS/MS, will be necessary to quantify individual bioactive constituents, including specific flavonoid glycosides and saponin congeners, and to validate the regulatory relationships suggested by our transcriptomic data. Fourth, this study focused on polysaccharides, flavonoids, and saponins, as these represent the three major bioactive classes of *P. sibiricum* with well-documented pharmacological significance. We acknowledge that other metabolites, such as alkaloids and phenolic acids, may also contribute to the overall pharmacological profile of this medicinal plant. However, the primary objective of this work was to dissect the regulatory mechanisms underlying the accumulation of these three principal component classes, which serve as the foundation for quality evaluation of this species. Future metabolomic investigations targeting a broader spectrum of metabolites will be valuable to fully characterize the bioactive landscape of different age nodes.

Despite these limitations, the present study establishes the first integrated chemical–transcriptomic framework for understanding age-dependent bioactive variation across individual rhizome nodes in *P. sibiricum*, providing both mechanistic insights into developmental metabolic reprogramming and practical guidance for precision harvesting and node-based quality classification.

## 4. Materials and Methods

### 4.1. Plant Materials

Rhizomes of four-year-old *Polygonatum sibiricum* Red. were collected in October 2024 from the Huangjing cultivation base of the Henan Academy of Agricultural Sciences and authenticated by Professor Huizhen Liang of the Institute of Chinese Herbal Medicines, Henan Academy of Agricultural Sciences. A total of 12 individual plants were sampled and have been deposited in the laboratory of the Institute of Chinese Herbal Medicines, Henan Academy of Agricultural Sciences.

Fibrous roots were removed, and the rhizomes were ultrasonically cleaned with deionized water. Based on the developmental characteristics of annual nodes, where each year corresponds to one node with a distinct stem scar on the internode, the rhizomes were sectioned from the proximal (younger) end to the distal (older) end according to the number of stem scars into four age classes, namely 1-year-old node (HJ1), 2-year-old node (HJ2), 3-year-old node (HJ3), and 4-year-old node (HJ4). The fresh weight of each age node was recorded using an electronic balance. The fresh weight of each age node was recorded using an electronic balance. The samples were then divided into two portions: one portion was immediately frozen with dry ice and stored at −80 °C for subsequent RNA extraction, and the other portion was placed in a forced-air drying oven at 108 °C for 30 min for enzyme inactivation, dried at 55 °C to constant weight, and pulverized for phytochemical analyses.

### 4.2. Bioactive Content Assay

Samples were placed in a forced-air drying oven at 108 °C for 30 min for enzyme inactivation, then dried at 55 °C to constant weight and pulverized for subsequent analyses.

The polysaccharide content was determined using the total polysaccharide content assay kits (JC0410-M, Nanjing Jice Biotechnology Co., Ltd., Nanjing, China) based on the phenol-sulfuric acid method with D-glucose as the standard. In brief, polysaccharides were extracted with water, precipitated with ethanol, and collected by centrifugation. Then, 1.0 mL of the sample solution was mixed with 1.0 mL of 5% phenol and 5.0 mL of concentrated sulfuric acid. After mixing, the reaction was incubated at 30 °C for 20 min, and the absorbance was measured at 490 nm.

The flavonoid content was determined according to the standard NY/T 1295-2007 [41] using the aluminum chloride colorimetric method with rutin as the standard. Samples were ultrasonically extracted with 70% ethanol. Then, 1.0 mL of the extract was sequentially mixed with 0.3 mL of 5% NaNO_2_ (reacted for 6 min), 0.3 mL of 10% AlCl_3_·6H_2_O (reacted for 6 min), and 2.0 mL of 1 M NaOH. The mixture was diluted to 5 mL with 70% ethanol, allowed to stand for 15 min, and the absorbance was measured at 510 nm.

The saponin content was measured using the total saponin content assay kit (JC2201-M, Nanjing Jice Biotechnology Co., Ltd., Nanjing, China) strictly following the manufacturer’s instructions, based on the vanillin-sulfuric acid method with oleanolic acid as the standard. Briefly, 0.2 mL of the sample solution (70% ethanol extract) was evaporated to dryness at 60 °C, followed by sequential addition of 0.2 mL of 5% vanillin-glacial acetic acid and 0.8 mL of perchloric acid. The mixture was heated at 60 °C for 15 min, cooled in an ice bath, and then 5.0 mL of glacial acetic acid was added. The absorbance was measured at 550 nm.

### 4.3. RNA Extraction and cDNA Library Construction for Illumina Sequencing

For transcriptomic analysis of samples across different plant age nodes, total RNA was first extracted using TRIzol Reagent (Thermo Fisher Scientific, Waltham, MA, USA), and mRNA was subsequently enriched using mRNA Capture Beads. Following purification, the mRNA was fragmented under high-temperature conditions and used as a template for the synthesis of first- and second-strand cDNA. The resulting cDNA underwent end repair, A-tailing, adapter ligation, and PCR amplification of the ligation products. The constructed transcriptome libraries were built on the Illumina NovaSeq X Plus platform (Beijing, China).

### 4.4. De Novo Assembly, Function Annotation, and Identification of Differentially Expressed Genes (DEGs)

Raw sequencing reads were processed using fastp (v0.18.0) to remove adapter sequences, reads with more than 10% ambiguous nucleotides (N), reads consisting entirely of adenine (A) bases, and low-quality reads wherein over 50% of bases had a quality score ≤ 20. The resulting clean reads were assembled de novo into unigenes using Trinity (v2.0). For functional annotation, these unigenes were searched against the NR, Swiss-Prot, KEGG, and COG/KOG databases via BLASTx (BLAST+ 2.13.0) with an E-value threshold of 1 × 10^−5^. Protein domain annotation was further performed using Pfam_Scan (v 1.6) against the Pfam database.

The assembled unigenes served as the reference transcriptome. Clean reads were then mapped back to these reference unigenes using Bowtie 2, and transcript abundances were quantified using RSEM (v1.3.0). After normalization for sequencing depth and transcript length, expression levels were calculated as fragments per kilobase of transcript per million mapped reads (FPKM). Differentially expressed genes (DEGs) were identified using DESeq2 (v1.30.0), with significance defined as |log_2_ (fold change)| ≥ 1 and false discovery rate (FDR) < 0.05. Functional enrichment of the DEGs was assessed against the Gene Ontology (GO) and KEGG databases using a hypergeometric test, with FDR < 0.05 as the significance threshold.

### 4.5. Real-Time Quantitative PCR (RT-qPCR) Validation

Total ribonucleic acid (RNA) was extracted using the Plant RNA Kit (Omega Bio-Tek, Norcross, GA, USA). The complementary deoxyribonucleic acid (cDNA) template was synthesized with HiScript III All-in-one RT SuperMix Ideal for quantitative polymerase chain reaction (qPCR) (+gDNA wiper) (Vazyme, Nanjing, China). The expression levels of target genes were assessed using a StepOnePlusTM Detection System (Applied Biosystems, Foster City, CA, USA). 18S ribosomal RNA was employed as a reference gene to normalize the expression data [14]. Primers were designed using Primer Premier 5, and the details are presented in Supplementary Table S9. Relative messenger RNA (mRNA) expression levels were calculated according to the 2^−ΔΔCt^ method [42].

### 4.6. Statistical Analysis

Statistical analyses of all biochemical indicators were performed using SPSS software V.19.0. One-way ANOVA followed by Duncan’s multiple comparisons test was used to analyse differences among groups, with a *p* value < 0.05 considered statistically significant. Pearson’s correlation coefficient |r| ≥ 0.5 and *p* value < 0.05 were employed for the correlation analysis.

## 5. Conclusions

In summary, this study systematically dissected the age-dependent chemical and transcriptional landscapes of four distinct rhizome nodes (HJ1–HJ4) within 4-year-old *P. sibiricum*, revealing that polysaccharides, flavonoids, and saponins accumulate asynchronously rather than following a uniform upward trajectory with increasing node age. Through comparative transcriptomic profiling, we identified 19,638 DEGs and demonstrated that a coordinated transcriptional reprogramming—characterized by early activation of backbone-construction genes followed by progressive upregulation of modification-and-decoration genes—operates across developmental stages, yet manifests through pathway-specific logic that accounts for the distinct accumulation peaks of each bioactive class. These findings challenge the conventional “older is better” assumption and provide a molecular framework for understanding how *P. sibiricum* rhizomes partition metabolic resources across developmental time. From a practical perspective, the node-specific chemical signatures support a targeted utilization strategy: HJ3 for polysaccharide-based pharmaceuticals, HJ4 for antioxidant-enriched functional products, HJ1 for saponin extraction, and HJ2 for bulk raw materials, thereby enabling a shift from indiscriminate harvesting to precision, value-oriented processing. While the present study is limited to a single genotype, location, and harvest year, the integrated chemical–transcriptomic approach established here lays a robust foundation for future multi-environment, multi-variety investigations and functional validations, ultimately contributing to the establishment of node-based quality grading standards and the sustainable, high-value utilization of *P. sibiricum* resources.

## Data Availability

The raw sequencing data generated in this study are available from the corresponding authors upon reasonable request.

## Supplementary Materials

The following supporting information can be downloaded at https://www.mdpi.com/article/10.3390/ijms27188282/s1.
